# S1-1 Whole systems approaches to the promotion of school-based physical activity

**DOI:** 10.1093/eurpub/ckad133.004

**Published:** 2023-09-11

**Authors:** Chair(s):, Anna Chalkley

**Affiliations:** Western Norway University of Applied Sciences, UK

## Abstract

Physical inactivity increases the risk of physical and mental ill-health and lower academic performance during childhood and is therefore a key public health priority. Whole-school approaches (WSA) are recommended as part of a system-wide approach to tackling physical inactivity. WSAs focus on aligning school policies, environments, school stakeholders and opportunities for physical activity within and beyond the school day. Yet, review-level evidence deems current WSAs ineffective due to: a lack of understanding of what constitutes a whole-school physical activity approach, programmes not considering schools as complex adaptive systems, limited multi-stakeholder input in programme design and poor use of implementation theory within programme design and delivery. Therefore, initiatives fail to overcome the systemic barriers affecting the adoption and development of new school practices. As a result, schools fail to adapt unique, context-specific policies to address environmental and stakeholder needs and are insufficient to improve children’s physical activity.

This symposium will present applied research relevant to the practice of whole systems approaches to whole school physical activity promotion in different European country contexts. In doing so, relevant work which addresses the interrelationships within and across levels of influence of the system will be presented, with the implications for practice, research and policy in the context of future challenges in this area, discussed.

